# Licensing microgels prolong the immunomodulatory phenotype of mesenchymal stromal cells

**DOI:** 10.3389/fimmu.2022.987032

**Published:** 2022-08-18

**Authors:** Matthew D. Patrick, Ramkumar T. Annamalai

**Affiliations:** Department of Biomedical Engineering, University of Kentucky, Lexington, KY, United States

**Keywords:** microgel, interferon gamma, licensing, mesenchymal stromal cells, MSC, gelatin

## Abstract

Mesenchymal stromal cells (MSC) are sensors of inflammation, and they exert immunomodulatory properties through the secretion of cytokines and exosomes and direct cell-cell interactions. MSC are routinely used in clinical trials and effectively resolve inflammatory conditions. Nevertheless, inconsistent clinical outcomes necessitate the need for more robust therapeutic phenotypes. The immunomodulatory properties of MSC can be enhanced and protracted by priming (aka licensing) them with IFNγ and TNFα. Yet these enhanced properties rapidly diminish, and prolonged stimulation could tolerize their response. Hence a balanced approach is needed to enhance the therapeutic potential of the MSC for consistent clinical performance. Here, we investigated the concentration-dependent effects of IFNγ and TNFα and developed gelatin-based microgels to sustain a licensed MSC phenotype. We show that IFNγ treatment is more beneficial than TNFα in promoting an immunomodulatory MSC phenotype. We also show that the microgels possess integrin-binding sites to support adipose tissue-derived MSC (AD-MSC) attachment and a net positive charge to sequester the licensing cytokines electrostatically. Microgels are enzymatically degradable, and the rate is dependent on the enzyme concentration and matrix density. Our studies show that one milligram of microgels by dry mass can sequester up to 641 ± 81 ng of IFNγ. Upon enzymatic degradation, microgels exhibited a sustained release of IFNγ that linearly correlated with their degradation rate. The AD-MSC cultured on the IFNγ sequestered microgels displayed efficient licensing potential comparable to or exceeding the effects of bolus IFNγ treatment. When cultured with proinflammatory M1-like macrophages, the AD-MSC-seeded on licensing microgel showed an enhanced immunomodulatory potential compared to untreated AD-MSC and AD-MSC treated with bolus IFNγ treatment. Specifically, the AD-MSC seeded on licensing microgels significantly upregulated *Arg1*, *Mrc1*, and *Igf1*, and downregulated *Tnfα* in M1-like macrophages compared to other treatment conditions. These licensing microgels are a potent immunomodulatory approach that shows substantial promise in elevating the efficacy of current MSC therapies and may find utility in treating chronic inflammatory conditions.

## Introduction

Mesenchymal stromal cell (MSC) therapy has attracted clinicians and researchers as a viable treatment option for many inflammatory and immune diseases. MSC are a potent immunomodulatory tool that can regulate innate and adaptive immune responses through direct cell-cell and paracrine signaling (1). Their potential to modulate disease states has been widely recognized, and there were over 900 clinical trials between 2004 and 2018 in which MSC were the primary disease-modifying agent (2). Further, at least 10 MSC-based therapies are approved globally for inflammatory and immune diseases such as graft-versus-host disease, Crohn’s disease, amyotrophic lateral sclerosis (ALS), and myocardial infarction (3). Although their effectiveness in treating certain diseases has been well documented, the outcomes are inconsistent and vary with the treatment timing and the inflammatory milieu (4).

MSC are sensors and modifiers of the body’s immune response, regulating the expression of specific cytokines, chemokines, and growth factors. During low inflammatory states, MSC adopt a quiescent phenotype with low prostaglandin E2 (PGE2) and HLA-G expression that are vital for sustaining T_H_1 and T_H_17 proliferation (5, 6). But during inflammation, MSC are activated or adopt a ‘licensed’ phenotype resulting in increased expression of immunomodulatory factors, including indolamine-2,3-dioxygenase (IDO, in humans), nitric oxide (NO, in mice), PGE2, TNFα-stimulated gene-6 (TSG6), transforming growth factor- β (TGF-β), IL-10, and IL-1RA (7). These factors suppress T_H_1 and T_H_17 cells and promote immunosuppressive T_regs_, thereby modulating the local inflammatory macrophages toward a pro-healing phenotype (8). It should be noted that the degree of MSC activation and subsequent modulation is context-specific and varies with the MSC tissue source and the changing disease state. Although these disease-modifying properties are beneficial for treating benign inflammatory conditions, their usefulness in chronic conditions is restrictive and inconsistent.

The efficacy of MSC therapies remains highly variable and is greatly affected by factors such as treatment timing, administration route, and hemocompatibility. In the clinic, MSC are administered locally or systemically, dependent on the clinical indication and pathology. But there is no clear consensus on the optimal mode of delivery (9). When administered locally, the MSC are concentrated at the injection site for a short time before they are cleared into systemic circulation (3, 10). In comparison, the systemic route relies on the innate ability of some types of MSC to naturally ‘home’ to specific niches (11). Although, a majority of the injected cells get trapped in the capillary bed of the lungs (12). In both routes, the MSC can get into the systemic circulation, and hence there is a risk of forming blood clots due to their expression of procoagulant tissue factor TF/CD142 (13). The thromboembolic risk varies with the MSC source and can be somewhat minimized through anticoagulation therapies (14). Further, in conditions characterized by intermittent flare-ups, such as arthritis, the timing of the treatment is crucial for achieving a robust therapeutic response (4). These factors contribute to the overall inconsistent outcomes of current MSC therapies. Addressing these limitations can help improve the efficacy and the clinical relevance of MSC-based therapies.

Like immune cells, MSC can keep a ‘memory’ of stimulus for a shorter period after moving to new environments (15). Therefore strategies for pre-conditioning or pre-licensing MSC using factors, including cytokines, hormones, hypoxia, and matrix mechanics, are being explored to promote a consistent and sustained therapeutic phenotype. Notably, pre-licensing with either IFNγ or TNFα has been shown to improve the intensity of their immunomodulatory effects. Stimulation of the MSC IFNγ receptor or TNFα receptor activates the JAK/STAT pathway or NFκB pathway respectively and leads to the subsequent regulation of the immunomodulatory factors (16). In animal models, IFNγ-licensed MSC are able to ameliorate inflammatory diseases like GvHD and colitis (17, 18). Further, IFNγ licensing can protect MSC from T-cell-mediated apoptosis, which is more pronounced after cryopreservation and thawing (19). Similarly, TNFα-licensed MSC exhibit therapeutic effects in a mouse model of inflammatory bowel disease (20). Although these pre-licensing strategies show enhanced potential for immunomodulation for a short time, their response fades away rapidly *in vivo*. Hence there is a critical need for strategies that can prolong the immunomodulatory phenotypes of MSC *in vivo* and prevent their rapid clearance from the site of injection. We propose a material-based approach that can anchor the MSC to the site of injection and prolong their immunomodulatory phenotype through a localized and sustained release of licensing factors.

Material-based systems are used widely in applications that require a sustained release of biochemical factors and deliver therapeutic cells. For instance, bone tissue engineering approaches that release osteoinductive factors have already seen clinical application (21). Material-based systems are customizable and suitable to augment the therapeutic efficacy of cells and biomolecules. Here, we used a modular microgel system that can sequester licensing cytokines while providing a suitable surface for MSC attachment and proliferation. The enzymatic degradation of the microgel matrix by the MSC releases the licensing factors locally to sustain an immunomodulatory phenotype of MSC. The microgels are composed of biocompatible gelatin polypeptides containing RGD motifs facilitating integrin-mediated cell attachment (22). Further, the polypeptides are crosslinked with genipin, a naturally occurring crosslinker with good cytocompatibility, to enhance their mechanical strength (23). This crosslinking depletes the positively charged amines of the lysine residues imparting a net negative charge on the crosslinked polypeptides (24), allowing ionic sequestration of biomolecules with a positively charged domains (25). MSC can be cultured on the surface of these microgels sequestered with cytokines on the core matrix and injected into the injury site in a non-invasive manner. Attachment to the microgel will increase MSC retention at the injection site, while the sequestered cytokines will provide local and sustained delivery to maintain a licensed phenotype. Additionally, the microgel’s unique physical characteristics, including the shape and stiffness, make them ideal for promoting osteogenesis of MSC and subsequent integration with the host tissue.

Here, we characterized the physical and biochemical properties of the microgels and demonstrated their ability to ameliorate inflammatory conditions. We show that the microgels efficiently sequester IFNγ and release it upon degradation while readily supporting MSC attachment. We also show that the localized release of IFNγ promotes an immunomodulatory MSC phenotype efficiently. Further, we confirm their immunomodulatory phenotype through macrophage co-culture studies. In addition, we evaluated the osteogenic potential of the microgels to show that they naturally direct MSC toward an osteogenic line over a prolonged culture period. Overall, our microgels show substantial promise in elevating the efficacy of current MSC therapies and demonstrate an improved option to address persistent chronic inflammatory diseases.

## Methods

### Microgel fabrication

**Figure 1:**
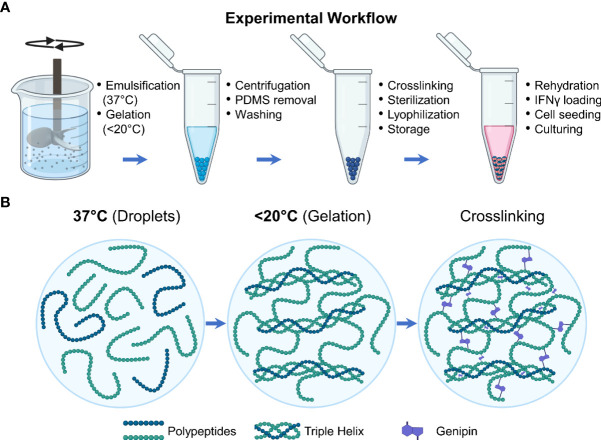
Experimental workflow. **(A)** Schematic showing the steps involved in the fabrication and preparation of the licensing microgels. **(B)** During the fabrication processes, the gelatin polypeptides undergo a temperature-driven structural reorganization which is solidified through genipin crosslinking.

Microgels were fabricated as previously described (**Figure 1A**) **(** 25). Briefly, gelatin derived from porcine skin (type A, 300 bloom, Sigma) was dissolved in deionized (DI) water to make a 6 wt% stock solution. The stock solution was dispensed dropwise into a polydimethylsiloxane (PDMS, viscosity = 100 cS, Clearco Products Co., Inc.) bath heated to 37°C and stirred by a propeller impeller at 500 rpm. After 5 min of emulsification, the PDMS bath was cooled with an ice bath for 30 min. They were then collected through a series of 275 g centrifugation and wash steps using Dulbecco phosphate buffer solution (DPBS, Invitrogen) supplemented with 1% TWEEN 20 (Sigma). The microgels were then crosslinked by adding 1 wt% genipin (Wako Chemicals) and left in an auto rotator for 48 hours. During these processes, the gelatin polypeptides undergo a temperature-driven structural reorganization which is solidified through genipin crosslinking to form the microgels (**Figure 1B**). The microgels were then washed with 100% ethanol to stop the crosslinking and remove excess genipin and swelled in DPBS for 24 hours. Finally, they were sonicated to remove aggregates and filtered to the desired size range using nylon mesh sieves.

### Size distribution and swelling rate

Bright-field imaging of microgels was done to measure the diameter and determine the size distribution of microgels. Microgels were dehydrated using 100% ethanol, flash-frozen in liquid nitrogen, and lyophilized overnight. Dehydrated microgels were rehydrated in DPBS, monitored using an inverted microscope, and recorded periodically. The images were then processed and analyzed using Fiji (ImageJ) software (National Institutes of Health) to determine the size and swelling rates.

### Enzymatic degradation

A stock solution was prepared by adding a precise amount of dehydrated and lyophilized microgels in 1X DPBS (stock = 0.67 mg/mL). 200 μL of the sock solution (~130 μg/well) was dispensed in a 96 well plate, and an equal volume of collagenase solution (Worthington Biochemical) was added to the wells at different concentrations and sealed with a clear plate cover to prevent evaporation. The fluorescence of the supernatant was then measured at Ex/Em 590/620 every 5 min for 48 hours to assess microgel degradation rates.

### Loading and release characteristics of microgels

For loading, 10 µL of IFNγ stock solution (100 µg/mL of DPBS) was added per 1 mg of lyophilized microgels and were placed in a humidified incubator for 24 hours. Then they were washed with 0.1 wt% bovine serum albumin (Sigma) in DPBS to remove unbound IFNγ. The maximum loading capacity was calculated by performing an ELISA assay on fully degraded microgels and measuring unbound IFNγ in the remaining solution after loading. To fully degrade the microgels and release the bound IFNγ, the loaded microgels were suspended in 1 mL of 100 U/mL collagenase solution and kept at 37°C until completely degraded. The supernatant was collected periodically and analyzed using an IFNγ ELISA kit to measure the release of IFNγ from the degrading microgel. The corresponding degradation rates of the microgels were also measured fluorometrically at Ex/Em 590/620.

### Isolation of primary adipose tissue-derived mesenchymal stromal cells

All studies involving animal cells or tissues are conducted according to approved protocols by the Institutional Biosafety Committee (IBC) protocol and Institutional Animal Care and Use Committee (IACUC, 2019-3346) at the University of Kentucky, respectively. Inguinal fat pads were isolated from adult mice (wildtype C57BL/6J - JAX stock #000664 and ACTb-EGFP expressing C57BL/6-Tg(CAG-EGFP)1Osb/J, JAX stock #003291), washed in serial dilutions of betadine and sterile 1X DPBS. The washed tissues were then finely minced with sterile scissors, and suspended in 0.1 wt% collagenase solution, and incubated at 37°C with constant agitation for 30 min or until most of the tissue was degraded. An equal amount of Dulbecco’s modified eagle’s medium (DMEM, Gibco) with 10% fetal bovine serum (FBS, Gibco) was then added to neutralize the collagenase and filtered through a 100 μm cell strainer. The single-cell suspension was then centrifuged at 250 g for 5 min, supernatant aspirated, and the cell pellet was resuspended in DMEM+10% FBS and cultured in a 6 well plate. After 24 hours, the wells were washed twice with warm 1x DPBS to remove non-adherent cells and debris leaving the adherent adipose tissue-derived mesenchymal stromal cells (AD-MSC). The cells were culture-expanded until the 5^th^ passage and used for experiments.

### AD-MSC culture and licensing

Primary AD-MSC were cultured in growth media containing DMEM+10% FBS+ antibiotics and antimycotics (Gibco). An IC-21 murine macrophage cell line (ATCC) was acquired, cultured in expansion media (RPMI (Gibco) + 10% FBS + antibiotics and antimycotic), and used for co-culture studies. All cells were maintained at 37°C in a CO_2_ incubator. To license AD-MSC, they were cultured until ~80 confluent in growth media and replaced with licensing media, i.e., growth media supplemented with the cytokines (TNFα, IFNγ, or both), for 24 hours. AD-MSC were seeded on microgels by incubating IFNγ loaded/unloaded microgels in growth media for 1 hour prior to seeding. This growth media was aspirated and replaced by 100 µL of MSC suspension (~0.3 million cells per mg of microgel) in growth media. The suspension was kept in the incubator for 1 hour to allow cell attachment to the surface of the microgels. During this period the suspension was gently mixed every 10 min to prevent aggregation and allow uniform seeding. The microgels were then washed with fresh growth media to remove unbound cells. For co-culture studies, macrophages were cultured in corresponding growth media until ~80% confluent and then polarized for 24 hours using cytokines (50 ng/mL LPS and 25 ng/mL IFNγ) suspended in growth media.

### Quantitative gene expression

For gene expression analysis, the samples were collected in TRIzol™ reagent (Invitrogen), and the whole RNA was isolated according to the manufacture’s protocol. A SuperScript™ III Platinum™ One-Step qRT-PCR Kit (ThermoFisher) and TaqMan probes (ThermoFisher) were used to analyze gene expression profiles using a QuantStudio 3 real-time PCR system (ThermoFisher). The following TaqMan probes were used for our studies: Gapdh (Mm99999915_g1) as a housekeeping control, Alp (Mm00475834_m1), Bglap (Mm03413826_mH), Col1a1 (Mm00801666_g1), Ibsp (Mm00492555_m1), Sp7 (Mm04209856_m1), Spp1 (Mm00436767_m1), Runx2 (Mm00501584_m1) for AD-MSC osteogenic analysis. Il-1β (Mm00434228_m1), Nos2 (Mm00440502_m1), Ccl2 (Mm00441242_m1), Tnfα (Mm00443260_g1), Lgals9 (Mm00495295_m1), Tgfβ (Mm01178820_m1), Il-6 (Mm00446190_m1), Ptgs2 (Mm00478374_m1), Pparγ (Mm01184322_m1), Arg1 (Mm00475988_m1), Cxcl2 (Mm00436450_m1), Mrc-1 (Mm00485148_m1), Il-12β (Mm00434174_m1), Igf1 (Mm00439560_m1) and, Chil3 (Mm00657889_mH). Gene expression data were presented as heatmaps showing foldchange (different treatment) or Z-scores (concentration and time-dependent change).

### Cell proliferation

Cell proliferation was analyzed by quantifying the total DNA content of samples using Quant-iT™ PicoGreen™ dsDNA Assay Kit (ThermoFisher). Lambda DNA standard was used for generating a calibration curve. Briefly, samples were collected at the specified time points and homogenized through sonication in 1X Tris-HCl buffer and centrifuged at 10,000 g for 10 min. The supernatant was then mixed with PicoGreen, incubated in the dark for 5 min, and the fluorescence was read at Ex/Em wavelength of 485/528 nm.

### Orthocresolphthalein complexone (OCPC) assay for calcium deposition

The o-cresolphthalein complex one (OCPC) method was used to quantify calcium deposition by the AD-MSC on microgels as previously described (26). Briefly, the samples were homogenized through sonication in 1X Tris-HCl buffer and centrifuged at 10,000 g for 10 min. The supernatant was then collected and mixed with a working solution containing 5% ethanolamine/boric acid buffer (Sigma), 5% o-cresolphthalein (Sigma), and 2% hydroxyquinoline (Sigma) in DI water. The mixture was incubated at room temperature in the dark for 10 min, and the absorbance was read at 575 nm. CaCl_2_ (Sigma) dissolved in DI water at different concentrations served as standards.

### Sircol™ soluble collagen assay

The collagen deposition by the AD-MSC was quantified using a Sircol soluble collagen assay kit (Biocolor). Briefly, the samples were homogenized through sonication in 1X Tris-HCl buffer and centrifuged at 10,000 g for 10 min. The supernatant was collected, and 20 μL of each sample was brought up to 100 μL with Tris-HCL buffer and then mixed with 0.5 mL of Sircol dye reagent. Samples were mixed for 30 min, centrifuged at 12,000 RPM for 10 min, the supernatant was decanted, and 750 μL of acid-salt wash reagent was added to the pellet. The suspension was mixed and centrifuged again at 12,000 RPM for 10 min, the supernatant was decanted, and 250 μL of alkali reagent was added to the pellet. This suspension was vortexed and incubated for 5 min at room temperature. The absorbance of the final solution was read at 555 nm. Rat-tail collagen was used to create a standard curve.

### Alkaline phosphatase activity assay

A commercially available ALP activity fluorometric assay kit (BioVision) was used according to the manufacturer’s protocol. Briefly, the samples were homogenized through sonication in Tris-HCl buffer and centrifuged at 10,000 g for 10 min. Then 100 μL of the supernatant was mixed with 20 μL of 0.5 mM 4-methylumbelliferyl phosphate (MUP) substrate and incubated at 25°C in the dark for 30 min. Finally, 20 μL of stop solution was added to the mixture, and fluorescence was measured at Ex/Em of 360/440 nm. Samples of 0.1-0.5 nmol of MUP substrate with purified ALP enzyme were used to create a standard curve.

### Immunofluorescence staining and confocal imaging

Samples were fixed in a 10% aqueous buffered zinc formalin (Z-Fix, Anatech) overnight, washed with 1X DPBS, and briefly incubated in a 0.1% Triton X-100 (Sigma) in 1X DPBS solution for 5 min before staining. Phalloidin conjugate (AF488, ThermoFisher) was used to stain the F-actin of the cell cytoskeleton and DAPI (Invitrogen) for staining the nucleus. Samples were stained for 30 mins in the dark and washed with 1X DPBS. Z-stacks of the stained samples were acquired using a Nikon A1R confocal microscope and processed using Fiji (ImageJ) software (NIH).

### Electron microscopy

Samples were fixed with a 3% glutaraldehyde in sodium cacodylate buffer overnight, washed with DI water, and incubated in 2% Osmium Tetroxide solution for 16 hours. Samples were washed with DI water and dehydrated using ethanol series. Samples were then flash-frozen in liquid nitrogen and lyophilized overnight. The dried samples were sputter-coated with a 5 nm thick layer of platinum using Leica Ace 500 sputter coater and imaged using an FEI Quanta 250 environmental scanning electron microscope (SEM).

### Statistics

All measurements were performed at least in triplicate. Data are plotted as means with error bars representing the standard deviation. The Pearson correlation coefficient (r) was used to evaluate the linear correlation between two variables. One- and Two-Way ANOVA were used to identify significance within groups, and Holms-Sidak pairwise comparisons followed to identify which groups were significant.

## Results

### Fabrication and characterization of microgels

**Figure 2:**
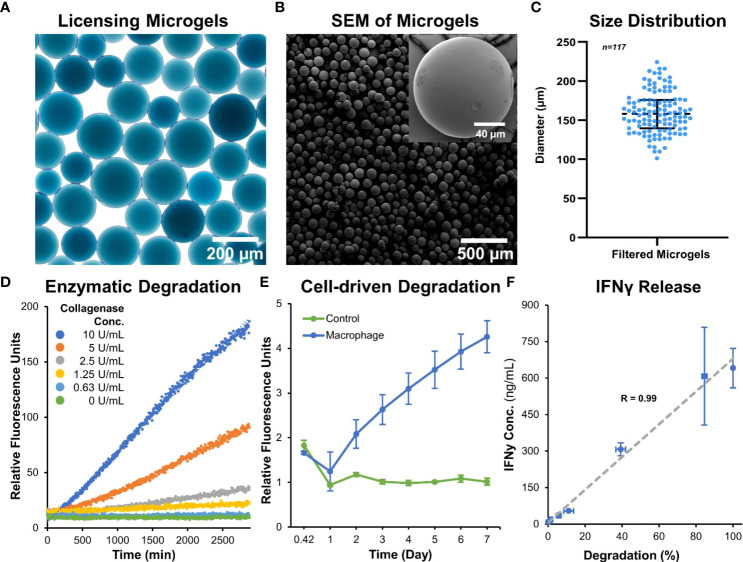
Microgel fabrication and characterization. **(A)** Bright-field image of sorted microgels show homogenous size distribution. **(B)** Scanning electron micrographs of dehydrated microgels show smooth surface and spherical morphology. **(C)** Size distribution of hydrated microgels. **(D)** Microgels exhibit a concentration-dependent degradation when incubated with collagenase solution. **(E)** A time-dependent degradation was seen when cultured with macrophages. **(F)** The release rate of IFNγ linearly correlated with the enzymatic-degradation of microgels (n = 4).

Microgels were fabricated, crosslinked for 48 hours in genipin, and separated to the desired size range of ~150 µm. This size range was chosen to provide sufficient surface for MSC attachment and spreading, while maintaining injectability for minimally invasive applications. The spherical morphology and homogeneous size distribution of the microgels (**Figures 2A-C**) allow them to conform to fill irregular wounds and articulating spaces. The microgels can be lyophilized (**Figure 2B** **)** and stored at room temperature for extended periods without any deterioration to their properties and can be rehydrated within seconds for cell delivery. When hydrated microgels were suspended in collagenase solution, they exhibited a concentration-dependent degradation (**Figure 2D**). Similarly, when microgels were cultured with macrophages, a time-dependent degradation was seen (**Figure 2E**). No degradation was seen in acellular control groups. The crosslinked microgel matrix possesses a net negative charge, as shown in the previous work (25), enabling them to sequester biomolecules with a positive charge. Since IFNγ has an isoelectric point (*pI*) of ~8.7 (27), they are suitable for ionic-complexation and sequestration in our microgels. We achieved a maximum loading of 641 ± 81 ng of IFNγ per mg of lyophilized microgel, further validated by the 309 ± 62 ng of unbound IFNγ measured in the remaining solution after loading. The release of IFNγ from the microgels depended on enzymatic degradation, and the release rate linearly correlated with the degradation rate (**Figure 2F**).

**Figure 3:**
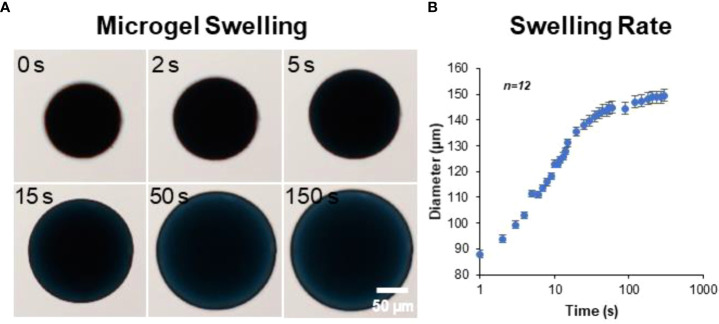
Microgel hydration and swelling. The microgels exhibited rapid swelling in an aqueous medium. **(A)** Time-lapse bright-field images showing microgel being hydrated in PBS. **(B)** The rate of hydration was rapid initially and plateaued within minutes to achieve an equilibrium state (n = 12).

The microgels also exhibit rapid swelling when suspended in an aqueous medium (**Figure 3A**). The rate of hydration was rapid initially and plateaued within minutes to achieve an equilibrium hydration condition (**Figure 3B**). An average percent increase in volume swelling ratio of ~400% was achieved in ~5 minutes.

### Licensing prolongs AD-MSC immunomodulatory phenotype

**Figure 4:**
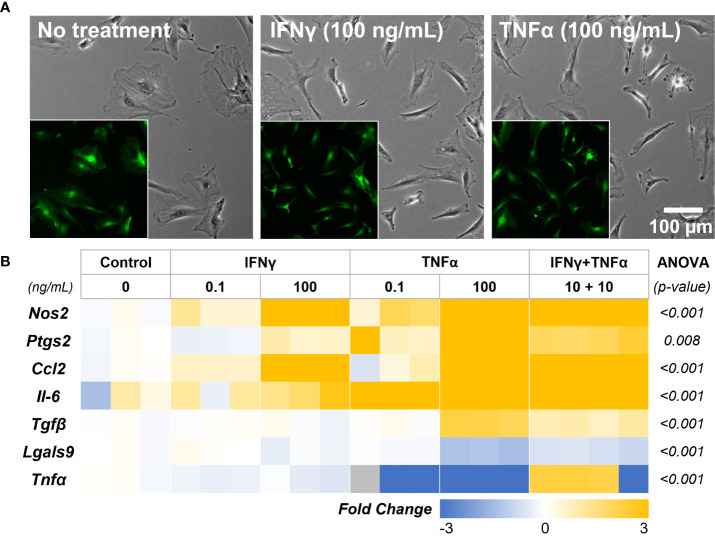
AD-MSC Licensing and their Immunomodulatory Phenotype. **(A)** Phase-contrast images of AD-MSC control and treatment conditions after 12 hours. (Insets) Corresponding fluorescent images of the cells show minimal differences in morphology and viability. **(B)** Heatmap of the AD-MSC gene expression after 12 hours of licensing using low and high concentrations of IFNγ and TNFα. Heatmaps show fold change with respect to the housekeeping gene (Gapdh) and untreated control.

We first investigated the concentration-dependent effects of two potent cytokines, IFNγ and TNFα, to sustain the immunomodulatory phenotype of adipose tissue-derived MSC (AD-MSC). A low (0.1 ng/mL) and high (100 ng/mL) concentration of the IFNγ and TNFα and a mixed treatment condition (10 ng/mL of each) were studied. AD-MSC that express green fluorescence protein conjugated to the actin cytoskeleton (ACTb-EGFP) were used to monitor changes in morphology and viability longitudinally (**Figure 4A**). No significant changes in morphology were seen in any treatment condition. However, the expression of *Nos2*, a key indicator of immunomodulatory phenotype, was significantly upregulated in all conditions (**Figure 4B**, See **Table 1** for groupwise comparison). *Ptgs2*, a precursor to prostaglandin E2 and a potent immunomodulatory cytokine, was significantly upregulated in the 100 ng/mL TNFα treatment. Inflammatory cytokines *Ccl2* and *Il-6* were also significantly upregulated in most cases, with the most significant effect seen in groups with TNFα treatment. Interestingly, *Il-6* expression was not significantly affected by the IFNγ treatment (*p>0.05*, **Table 1**). Immunoregulatory and angiogenic proteins *Tgfβ* and Lgals9 were significantly influenced by the 100 ng/mL TNFα treatment and combination groups, with *Lgals9* being downregulated and *Tgfβ* being upregulated. The TNFα treatment negatively influenced *Tnfα* expression, while no significant change was seen in the IFNγ treatment. The greatest increase in the most potent immunomodulatory factors (*Nos2* and *Ptgs2*) was seen in the TNFα treatment, but it also exhibited the highest upregulation of inflammatory cytokines (*Il-6* and *Ccl2*). IFNγ treatment, on the other hand, upregulated immunomodulatory factors while having modest effects on inflammatory factors. Hence, IFNγ is deemed more suitable for sustained licensing of AD-MSC.

**Table 1:** AD-MSC licensing through IFNγ and TNFα treatment.

Holm-Sidak Pairwise Comparison (vs. Control) *(p-value)*						
Genes	ANOVA *(p-value)*	IFNγ		TNFα		IFNγ + TNFα
*(ng/mL)*	-	0.1	100	0.1	100	10 + 10
***Nos2***	<0.001	0.024*	<0.001*	<0.001*	<0.001*	<0.001*
***Ptgs2***	0.008	0.77	0.673	0.094	0.011*	0.099
***Ccl2***	<0.001	0.307	<0.001*	0.584	<0.001*	<0.001*
***Il-6***	<0.001	0.605	0.274	<0.001*	<0.001*	<0.001*
***Tgfβ***	<0.001	0.923	0.751	0.95	<0.001*	<0.001*
***Lgals9***	<0.001	0.546	0.99	0.357	<0.001*	<0.001*
***Tnfα***	<0.001	0.797	0.947	<0.001*	0.003*	0.816

*indicates p < 0.05.

### IFNγ licensing retains AD-MSC phenotypes in a concentration-dependent manner

**Figure 5:**
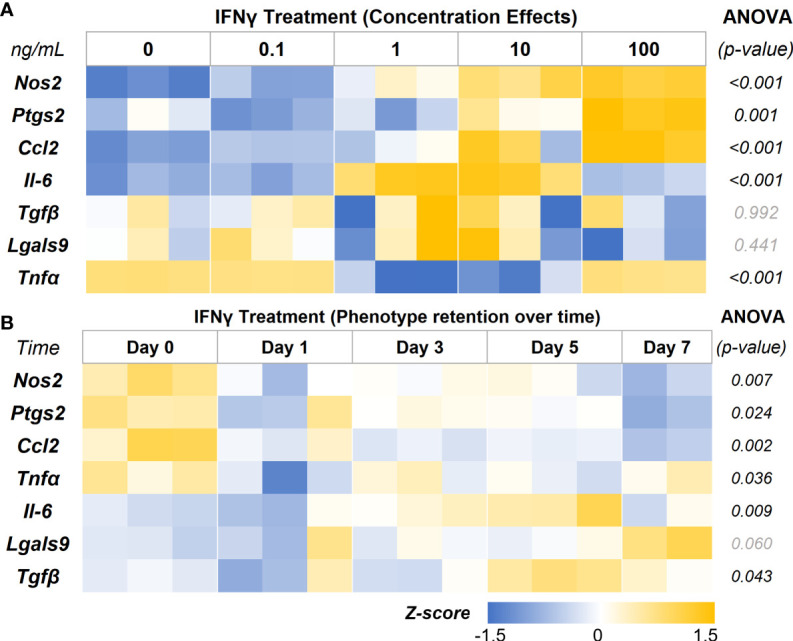
IFNγ Licensing and Retention of AD-MSC Immunomodulatory Phenotype. **(A)** Heatmap of the AD-MSC gene expression after 24 hours of licensing using different concentrations of IFNγ shows a concentration-dependent effect. **(B)** Heatmap of AD-MSC licensed for 24 hours using 10 ng/mL IFNγ showing robust phenotype retention over time.

In-depth screening of IFNγ treatment with a range of concentrations (0.1, 1, 10, and 100 ng/mL) showed a concentration-depended increase in the expression levels of the key immunomodulatory markers (**Figure 5A**, **Table 2**). The genes *Nos2*, *Ptgs2*, and *Ccl2* are upregulated in a concentration-dependent manner, with peak expression levels at 100 ng/mL treatment. Interestingly, *Il-6* expression was upregulated at 1 and 10 ng/mL, but no change was seen at 100 ng/mL. Likewise, *Tnfα* expression showed significant downregulation at 1 and 10 ng/mL, but no change was seen at 100 ng/mL. *Tnfα* and *Il-6* are potent proinflammatory cytokines that could negatively impact the regenerative milieu. Hence, we deemed concentrations from 10 and 100 ng/mL suitable for subsequent studies.

**Table 2:** IFNγ licensing is concentration-dependent – Groupwise comparison.

Holm-Sidak Pairwise Comparison (vs. Control) *(p-value)*					
Genes	ANOVA *(p-value)*	IFNγ			
*(ng/mL)*	-	0.1	1	10	100
***Nos2***	<0.001	0.014*	<0.001*	<0.001*	<0.001*
***Ptgs2***	0.001	0.129	0.367	0.164	<0.001*
***Ccl2***	<0.001	0.283	0.136	0.011*	<0.001*
***Il-6***	<0.001	0.53	<0.001*	<0.001*	0.168
***Tgfβ***	0.992	N/A	N/A	N/A	N/A
***Lgals9***	0.441	N/A	N/A	N/A	N/A
***Tnfα***	<0.001	0.872	<0.001*	<0.001*	0.979

N/A indicates no significance in ANOVA between groups. * indicate a significant change in gene expression.

Then we investigated the retention of the licensed AD-MSC phenotype over 7 days after the initial treatment. The AD-MSC were treated with 10 ng/mL of IFNγ for 24 hours, washed thoroughly to remove all cytokines, and monitored for 7 days (**Figure 5B**, **Table 3**). *Nos2*, one of the critical factors of the licensed phenotype, was downregulated after 24 hours. In contrast, *Ptgs2* expression remained consistent until day 7, when it was significantly downregulated, indicating a sustained phenotype. *Ccl2* expression dropped within 24 hours and continued to drop until day 7. *Tnfα* expression, on the other hand, showed downregulation after 24 hours but bounced back to licensed levels. *Il-6* expression remained unchanged until day 7, while *Lgals9 and Tgfb* did not show significant changes. Overall, the licensed phenotype of the AD-MSC were partially retained for a week after licensing signals were removed.

**Table 3:** AD-MSC phenotype retention after IFNγ licensing pairwise comparison.

Holm-Sidak Pairwise Comparison (vs. Control) *(p-value)*					
Genes	ANOVA *(p-value)*	Day 1	Day 3	Day 5	Day 7
***Nos2***	0.007	0.009*	0.023*	0.023*	0.004*
***Ptgs2***	0.024	0.077	0.151	0.132	0.009*
***Ccl2***	0.002	0.003*	0.002*	0.003*	<0.001*
***Tnfα***	0.036	0.019*	0.546	0.199	0.614
***Il-6***	0.009	0.678	0.150	0.776	0.011*
***Lgals9***	0.060	N/A	N/A	N/A	N/A
***Tgfβ***	0.043	0.774	0.871	0.078	0.679

N/A indicates no significance in ANOVA between groups. * indicate a significant change in gene expression.

### AD-MSC seeded on licensing microgels exhibit a sustained immunomodulatory phenotype

**Figure 6:**
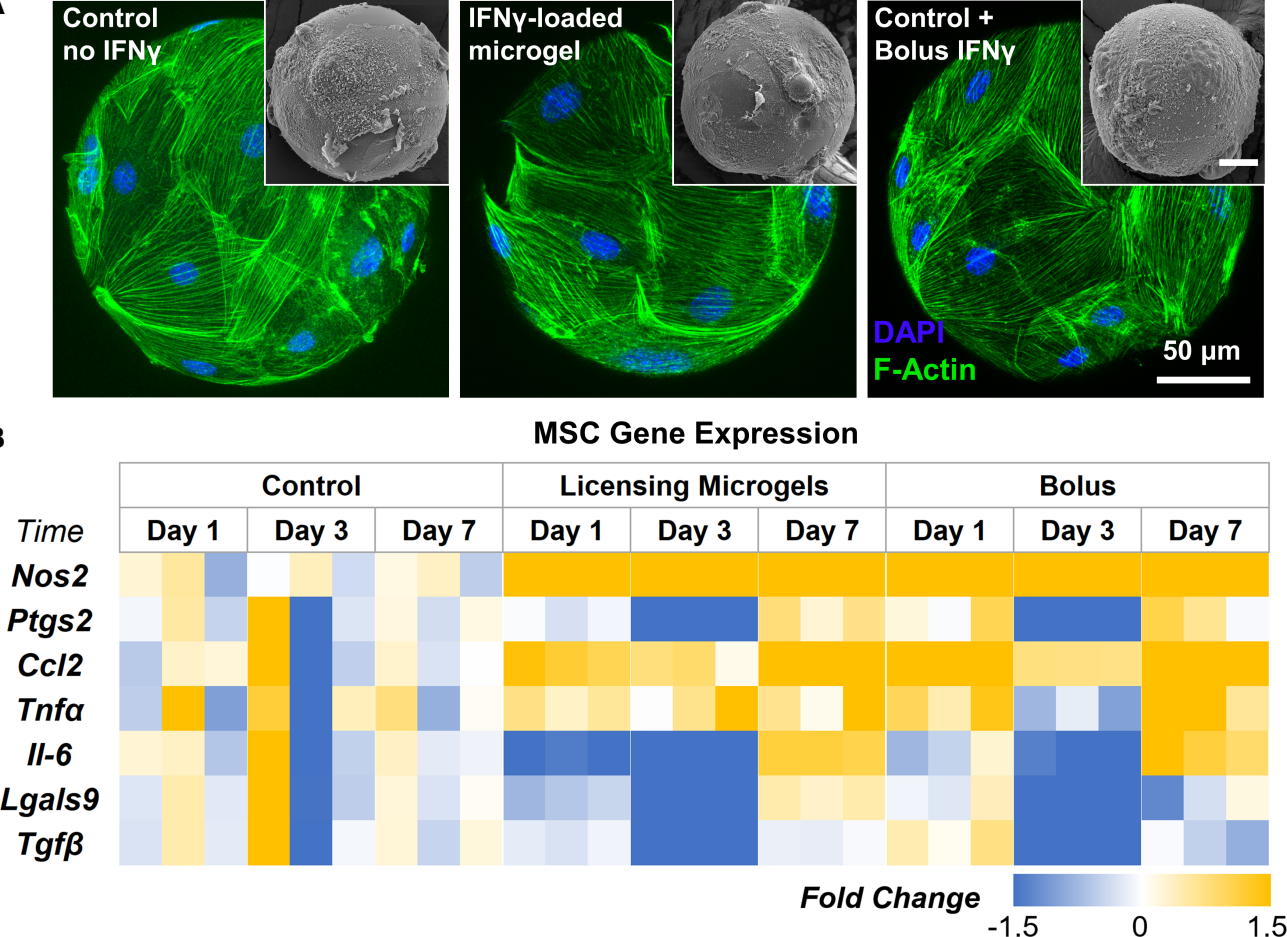
Gene Expression of AD-MSC Seeded on licensing Microgels. **(A)** Confocal Z-stacks of microgels seeded with AD-MSC (Control microgels, IFNγ loaded microgels, and IFNγ bolus treatment). (Insets) Corresponding SEM images of microgels. **(B)** Heatmap showing gene expression of inflammatory and anti-inflammatory AD-MSC phenotypes. Heatmaps show fold change with respect to the housekeeping gene (Gapdh) and no treatment control group.

AD-MSC seeded on the microgels rapidly attach and spread on the surface and fully cover them within 24 hours (**Figure 6A**). AD-MSC seeded on IFNγ-loaded microgels showed a similar morphology and viability and sustained a licensed phenotype for 7 days with a consistent expression of *Nos2* over time (**Figure 6B**, **Table 4**). The expression pattern for both the licensing microgels and the bolus treatment (IFNγ supplied in the media) groups was similar over 7 days, with minor differences in *Ccl2* and *Nos2* expression on days 1 and 7, respectively. Interestingly, both groups exhibited downregulation in *Ptgs2*, *Lgals9*, and *Tgfβ* on day 3 relative to the no treatment control, indicating the phenotype similarities between the treatment groups. *Il-6* was significantly downregulated on days 1 and 3 in the microgel group and only on day 3 in the bolus treatment group. No significant change in *Tnfα* expression was noticed in any conditions. Overall, the microgels enhanced and protracted the immunomodulatory phenotype of AD-MSC as effectively as the bolus group without needing constant replenishment of the licensing factors.

**Table 4:** Pairwise comparison of all Time Points within Conditions and all Conditions within Time Points.

Holm-Sidak Pairwise Comparison (Time Point within Condition) *(p-value)*									
	Control			Microgel			Bolus		
Genes	Day 1 vs Day 3	Day 1 vs Day 7	Day 3 vs Day 7	Day 1 vs Day 3	Day 1 vs Day 7	Day 3 vs Day 7	Day 1 vs Day 3	Day 1 vs Day 7	Day 3 vs Day 7
***Nos2***	>0.999	>0.999	>0.999	0.036*	0.035*	0.862	0.004*	0.095	0.12
***Ptgs2***	>0.999	>0.999	>0.999	0.002*	0.198	<0.001*	<0.001*	0.777	<0.001*
***Ccl2***	>0.999	>0.999	>0.999	0.274	0.014*	0.002*	0.014*	0.204	0.001*
***Tnfα***	NA	NA	NA	NA	NA	NA	NA	NA	NA
***Il-6***	>0.999	>0.999	>0.999	0.737	<0.001*	<0.001*	0.061	0.042*	<0.001*
***Lgals9***	>0.999	>0.999	>0.999	0.043*	0.086	0.001*	0.016*	0.4	0.067
***Tgfβ***	>0.999	>0.999	>0.999	0.004*	0.886	0.004*	<0.001*	0.083	0.008*
									
Holm-Sidak Pairwise Comparison (Condition within Time Point) *(p-value)*									
	Day 1			Day 3			Day 7		
Genes	Control vs Microgel	Control vs Bolus	Microgel vs Bolus	Control vs Microgel	Control vs Bolus	Microgel vs Bolus	Control vs Microgel	Control vs Bolus	Microgel vs Bolus
***Nos2***	<0.001*	<0.001*	0.117	<0.001*	<0.001*	0.54	<0.001*	<0.001*	0.012*
***Ptgs2***	0.785	0.73	0.702	0.001*	0.002*	0.997	0.661	0.547	0.958
***Ccl2***	0.083	0.001*	0.045*	0.517	0.498	0.799	<0.001*	<0.001*	0.669
***Tnfα***	NA	NA	NA	NA	NA	NA	NA	NA	NA
***Il-6***	0.037*	0.598	0.074	0.018*	0.041*	0.568	0.144	0.174	0.921
***Lgals9***	0.518	0.977	0.647	0.007*	0.012*	0.665	0.454	0.658	0.336
***Tgfβ***	0.676	0.626	0.501	0.002*	0.001*	0.848	0.784	0.731	0.76

N/A indicates no significance in ANOVA between groups. * indicate a significant change in gene expression.

### Licensed AD-MSC modulate phenotype of inflammatory macrophages

**Figure 7:**
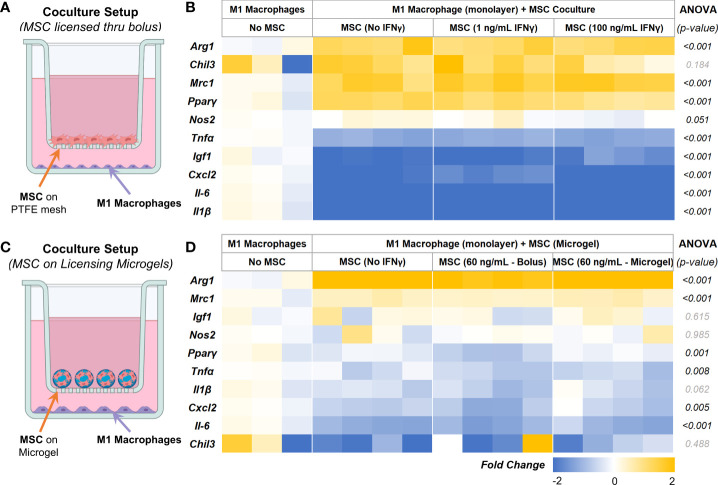
AD-MSC and M1 Macrophage Cocultures. **(A)** Schematic of the co-culture setup to evaluate the MSC paracrine effects on M1-like macrophages. **(B)** Heatmap showing the expression levels of proinflammatory and prohealing genes by macrophages after 24 hours of co-culture with licensed AD-MSC. **(C)** Schematic of the co-culture setup showing AD-MSC seeded on microgels in the trans-well and M1 macrophages on the bottom well. **(D)** Heatmap showing the expressional levels of proinflammatory and prohealing genes by macrophages after 24 hours of co-culture with AD-MSC on microgels. All fold changes are with respect to the housekeeping gene, Gapdh, and quiescent control AD-MSC.

To validate the immunomodulatory effects, AD-MSC seeded in 2D tissue culture plastics and microgels were licensed with bolus IFNγ and co-cultured with proinflammatory M1-like macrophages in trans-well plates, as shown (**Figure 7A**). All cells were washed thoroughly in PBS to remove all biochemical factors before initiating the co-culture. Serum-free media was used for co-culture studies. After 24 hours of co-culture, AD-MSC and macrophage samples were collected and analyzed individually. M1 macrophage monocultures served as controls. In all co-culture conditions, the AD-MSC modulated macrophages from an M1-like phenotype toward a proreparative M2-like phenotype (**Figure 7B**, **Table 5**). In general, the AD-MSC co-culture significantly upregulated M2 markers *Mrc1*, *Pparγ*, and *Arg1* and downregulated M1 markers *Il-6*, *Cxcl2*, *Tnfα*, and *Il-1β* in macrophages. But, *Pparγ* expression was upregulated at a relatively higher level when AD-MSC were licensed with 1 ng/mL than at 100 ng/mL IFNγ concentration. Likewise, *Cxcl2* was downregulated more prominently by AD-MSC licensed at 100 ng/mL IFNγ than the other treatment conditions (For pairwise comparisons, see **Table 5**).

**Table 5:** Pairwise comparison between all treatment conditions.

Holm-Sidak Pairwise Comparison (Between Groups) *(p-value)*							
Genes	ANOVA *(p-value)*	M1 vs Control	M1 vs 1 ng IFNγ	M1 vs 100 ng IFNγ	Control vs 1 ng IFNγ	Control vs 100 ng IFNγ	1 ng vs 100 ng IFNγ
***Arg1***	<0.001	<0.001*	<0.001*	<0.001*	0.809	0.846	0.770
***Chil3***	0.184	N/A	N/A	N/A	N/A	N/A	N/A
***Mrc1***	<0.001	<0.001*	<0.001*	<0.001*	0.469	0.480	0.832
***Pparγ***	<0.001	<0.001*	<0.001*	<0.001*	0.439	0.026*	0.071
***Nos2***	0.051	N/A	N/A	N/A	N/A	N/A	N/A
***Tnfα***	<0.001	<0.001*	<0.001*	<0.001*	0.745	0.553	0.496
***Igf1***	<0.001	<0.001*	<0.001*	<0.001*	0.643	0.027*	0.018*
***Cxcl2***	<0.001	<0.001*	<0.001*	<0.001*	0.004*	0.110	<0.001*
***Il-6***	<0.001	<0.001*	<0.001*	<0.001*	0.812	0.081	0.084
***Il1β***	<0.001	<0.001*	<0.001*	<0.001*	0.286	0.171	0.584

N/A indicates no significance in ANOVA between groups. * indicate a significant change in gene expression.

Then we investigated the effects of AD-MSC licensed using IFNγ sequestered on microgels on M1 macrophages and how they compare to other treatment groups. The microgels seeded with AD-MSC were separated from the M1 macrophages using transwells, as shown (**Figure 7C**). We used 1/10^th^ of the maximum loading capacity of the microgels (60 ng of IFNγ/mg of lyophilized microgels) and evaluated their potential in modulating AD-MSC phenotype and function using co-culture studies. We compared the effects of the licensing microgels to an equivalent bolus media (60 ng/mL of IFNγ) treatment that is renewed every other day. Untreated AD-MSC served as controls. In all co-culture conditions, the AD-MSC were seeded on the microgels, as shown (**Figure 7C**). As seen with monolayer cultures, the AD-MSC seeded on all microgel groups modulated the M1 macrophages towards a proreparative M2-like phenotype. Notably, the IFNγ sequestered microgels outperformed both the untreated control and bolus treatment in downregulating the inflammatory cytokine *Tnfα* in macrophages (**Figure 7D**). Similar levels of modulatory effects were seen in terms of upregulating *Arg1* and *Mrc1* and downregulating *Cxcl2* and *Il-6* between the bolus and the IFNγ sequestered microgel groups (For pairwise comparisons, see **Table 6**). Overall, these data show the notable effects of the IFN γ sequestered microgels in enabling and sustaining the AD-MSC immunomodulatory phenotype in macrophage co-cultures.

**Table 6:** Pairwise comparison between all treatment conditions.

Holm-Sidak Pairwise Comparison (Between Groups) *(p-value)*							
Genes	ANOVA *(p-value)*	M1 vs AD-MSC-Unlicensed	M1 vs AD-MSC-Licensed (Bolus)	M1 vs AD-MSC-Licensed (Microgel)	AD-MSC-Unlicensed vs AD-MSC-Licensed (Bolus)	AD-MSC-Unlicensed vs AD-MSC-Licensed (Microgel)	AD-MSC-Licensed (Bolus) vs AD-MSC-Licensed (Microgel)
***Arg1***	<0.001	<0.001*	<0.001*	<0.001*	0.006*	0.108	0.082
***Mrc1***	<0.001	<0.001*	0.004*	<0.001*	0.419	0.883	0.431
***Igf1***	0.615	N/A	N/A	N/A	N/A	N/A	N/A
***Nos2***	0.985	N/A	N/A	N/A	N/A	N/A	N/A
***Pparγ***	0.001	0.318	0.002*	0.532	0.011*	0.507	0.004*
***Tnfα***	0.008	0.207	0.014*	0.014*	0.209	0.183	0.887
***Il1β***	0.062	N/A	N/A	N/A	N/A	N/A	N/A
***Cxcl2***	0.005	0.039*	0.004*	0.085	0.269	0.552	0.147
***Il-6***	<0.001	<0.001*	<0.001*	<0.001*	0.978	0.739	0.609
***Chil3***	0.488	N/A	N/A	N/A	N/A	N/A	N/A

N/A indicates no significance in ANOVA between groups. * indicate a significant change in gene expression.

## Discussion

MSC are the sensors of the body’s immune response, can be derived from various adult tissue sources, and possess immune modulatory functions. They can promote inflammation when the immune response is underactive and restrain the inflammatory response when it is overactive. The immunomodulatory effects of MSC vary depending on their tissue source and activation state. The major immunomodulatory factors secreted by MSC include NO, PGE2, TGFβ, Galactin 9, and TSG6 (8). In our studies, we freshly isolated adipose tissue-derived MSC (AD-MSC) from inguinal fat pads because of the clinical relevance of the tissue source. When the AD-MSC were licensed with IFNγ or TNFα, they consistently upregulated a set of markers, including *Nos2*, *Ccl2*, and *Il-6*. *Nos2* was the most highly upregulated gene which results in the production of nitric oxide and subsequent suppression of T-cell proliferation (28). *Ccl2*, aka monocyte chemoattractant protein-1, is generally responsible for mobilizing monocytes, memory T lymphocytes, and natural killer cells to initiate inflammatory response (29). But, studies using murine bone marrow MSC found that *Ccl2* polarizes inflammatory macrophages toward regenerative (IL10-secreting) macrophages and treats inflammatory colitis (30). Notably, when TNFα alone was used for licensing, *Il-6* was highly upregulated in MSC. IL-6 is an inflammatory cytokine released in response to infection or tissue damage, triggering an inflammatory response from regulatory cells. They also play a crucial role in directing T_H_17 differentiation on the T_H_17/T_reg_ axis (31). Further, the expression of *Ptgs2*, necessary for PGE2 enzyme synthesis, is positively influenced only when AD-MSC were treated with IFNγ and not with TNFα treatment. Due to the elevated expression of *Il-6* by AD-MSC in all treatment formulations containing TNFα, IFNγ was chosen as the best licensing candidate for our studies. Further, IFNγ licensing can augment the immunosuppressive potency of MSC even after cryopreservation (32). They can also protect MSC from T-cell-mediated apoptosis, to which they are more susceptible after cryopreservation and thawing (19). This is critical for clinical application as cryopreservation simplifies the logistic of MSC-based cell therapy. IFNγ showed concentration-dependent licensing effects on AD-MSC, with low concentrations (<10 ng/mL) showing minimal licensing effects with high expression of *Il-6.* But at medium to high concentrations (10-100 ng/mL), they promoted a robust immunomodulatory AD-MSC phenotype where *Ptgs2* is upregulated, and *Tnfα* is downregulated. Over time, some licensed phenotypes, notably, *Nos2* and *Ccl2* expressions, diminish if the media is not replenished with IFNγ. Then we tested the ability of the licensing microgels to prolong a licensed-MSC phenotype.

The unique physical and chemical properties of gelatin microgels make them versatile carriers for cell and growth factor delivery. They can form complexes with different drugs, cytokines, and growth factors and are therefore widely used in drug delivery and tissue engineering applications (33). The tunable properties of the gelatin matrix, such as the crosslinking density and the isoelectric point, enable the optimization of degradation and drug delivery kinetics. The microscale nature of the microgels we fabricated allows them to be injected minimally invasively using hypodermic needles and conformally fill various tissue defects. Further, the microgels can be lyophilized and stored at room temperature for an extended period without degradation or deformation. This property enables off-the-shelf use, which is desirable for clinical applications. Our studies show that the enzymatic degradation of microgels is concentration-dependent and hence can allow delivery of sequestered factors in a bioresponsive manner, particularly in inflammatory conditions like osteoarthritis, where there are elevated levels of matrix metalloproteases (MMPs) during flare-ups (34). Hence the microgels can titer a therapeutic response based on the disease state. Further, we show that the degradation of the microgels and the release of sequestered IFNγ from the microgels are linearly correlated, indicating a homogenous polymer density and cytokine sequestration. For our cell culture studies, the average size of the microgels was kept at 150 μm in diameter, large enough to allow MSC to attach and spread on the surface and injectable when needed. The high hydration and the swelling rate (>400% volume increase) of the microgels also show cytocompatibility, which can be tailored by varying the crosslinking density. The AD-MSC readily attached and spread on the surface of the microgels. As expected, the loaded microgels were able to sustain the established licensed MSC characteristics over 7 days at a similar level to that of the bolus control. The IFNγ sequester by the microgels is slowly released as the MSC, known to secrete MMP (35), degrades the gelatin. This sustained licensed state of MSC is critical to addressing chronic or reoccurring inflammation and can help improve the efficacy of MSC therapies.

To confirm the therapeutic effects of the licensed AD-MSC, co-culture studies were performed using IFNγ licensed AD-MSC and M1-like macrophages. As expected, the licensed AD-MSC were able to modulate the inflammatory macrophage phenotype toward a reparative M2-like phenotype. Similar co-culture studies using bone marrow-derived MSC also show downregulation of IL-6 and TNFα release in inflammatory macrophages (36). Other material-based approaches involving immobilization of IFNγ have also shown success in ameliorating T-cell-induced inflammatory reponse (37). Notably, in our study, when AD-MSC were licensed using microgels, they upregulated *Arg1*, *Mrc1*, and *Igf1* in macrophages more robustly compared to monolayer cultures. These enhancements can be attributed to the tissue-like microenvironment provided by the microgels. It is known that substrate mechanics influence MSC phenotype, including their immunomodulatory capabillites (38). In addition, the licensed AD-MSC significantly downregulated *Tnfα* compared to the unlicensed AD-MSC. In the absence of any licensing cytokines, the microgels also supported AD-MSC osteogenesis (See **Supplementary Figure 1** and **Supplementary Table 1**), presumably due to their spherical morphology, which is known to promote MSC osteogenesis (39, 40).

In conclusion, we show that microgels efficiently sequester IFNγ and release it upon degradation while readily supporting MSC attachment. We also show that the localized release of IFNγ licenses the AD-MSC efficiently and further confirm their immunomodulatory phenotype through macrophage co-culture studies. Overall, licensing AD-MSC using the microgel system is a simple and effective tool to sustain the immunomodulatory properties of MSC and enhance their therapeutic applications in various instances.

## Data availability statement

The original contributions presented in the study are included in the article/**Supplementary Material**. Further inquiries can be directed to the corresponding author.

## Ethics statement

All studies involving animals cells or tissues are conducted according to approved protocols by the Institutional Biosafety Committee (IBC) protocol and Institutional Animal Care and Use Committee (IACUC) at the University of Kentucky.

## Author contributions

MP and RT contributed to the conception and design of the study, performed the statistical analysis, and wrote the manuscript. MP performed all experiments and data acquisition. All authors contributed to manuscript revision, read, and approved the submitted version.

## Funding

Research reported in this publication was supported in part by the National Institute of Arthritis and Musculoskeletal and Skin Diseases (NIAMS) Award Number R21AR078447, National Institute of General Medical Sciences (NIGMS) of the National Institutes of Health under Award Numbers P20GM130456 and P20GM103436-20 (KY IDeA Networks of Biomedical Research Excellence), National Center for Research Resources and the National Center for Advancing Translational Sciences of the National Institutes of Health under Award Number UL1TR001998, and Orthopedic Trauma Association (OTA, Grant Number: 6889). The content is solely the responsibility of the authors and does not necessarily represent the official views of the National Institutes of Health or other grant funding agencies.

## Acknowledgments

The authors would also like to thank Dr. Eric Blalock in the University of Kentucky Department of Pharmacology and Nutritional Sciences for consultation and advice on statistical analysis of the gene expression data.

## Conflict of interest

The authors declare that the research was conducted in the absence of any commercial or financial relationships that could be construed as a potential conflict of interest.

## Publisher’s note

All claims expressed in this article are solely those of the authors and do not necessarily represent those of their affiliated organizations, or those of the publisher, the editors and the reviewers. Any product that may be evaluated in this article, or claim that may be made by its manufacturer, is not guaranteed or endorsed by the publisher.
