# Supplementary material for: Licensing microgels prolong the immunomodulatory phenotype of mesenchymal stromal cells
Source: Front Immunol. 2022 Aug 18;13:987032. doi: 10.3389/fimmu.2022.987032 (PMC9433901; doi:10.3389/fimmu.2022.987032)
Supplement: Supplementary file 1 [file DataSheet_1.docx]

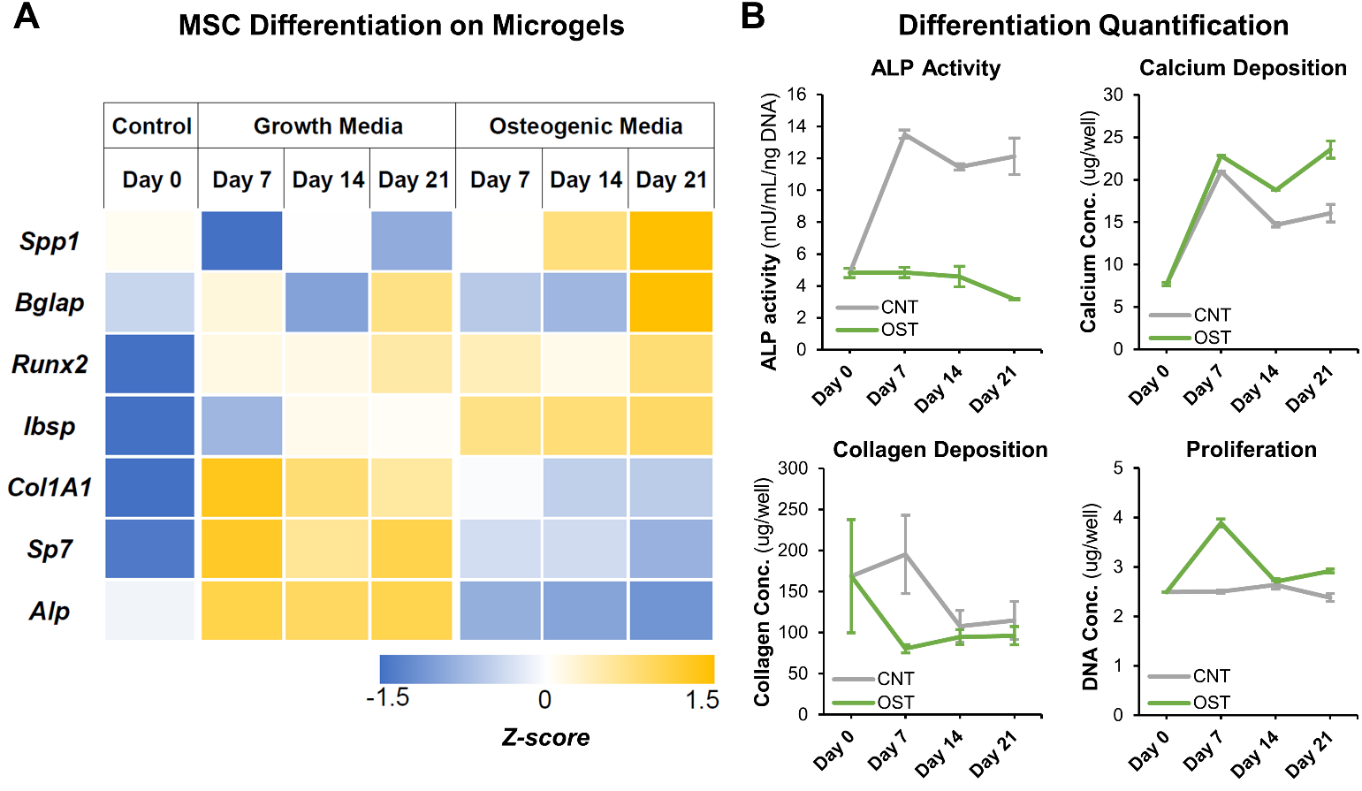


Supplementary Fig 1. Osteogenic Gene Expression and Characterization. **A)** Fold change of common osteogenic markers when AD-MSC are seeded on microgels with growth and osteogenic differentiation media. **B)** Quantification of ALP activity and calcium deposition.

Supplementary Table 1. Pairwise comparison of all treatment conditions with control. p(-1.65>Z>1.65) <0.05

| **Pairwise Z-Score** (vs. Control) | | | | | | |
| --- | --- | --- | --- | --- | --- | --- |
|  | **Growth Media** | | | **Osteogenic Media** | | |
| **Genes** | **Day 7** | **Day 14** | **Day 21** | **Day 7** | **Day 14** | **Day 21** |
| ***Spp1*** | -1.58 | -0.09 | -0.97 | -0.06 | 0.69 | 1.46 |
| ***Bglap*** | 0.65 | -0.54 | 1.16 | -0.12 | -0.33 | 2.27* |
| ***Runx2*** | 2.36* | 2.34* | 2.72* | 2.62* | 2.32* | 3.01* |
| ***Ibsp*** | 1.06 | 1.96* | 1.89* | 2.56* | 2.64* | 2.75* |
| ***Col1A1*** | 2.98* | 2.45* | 2.21* | 1.60 | 1.14 | 1.10 |
| ***Sp7*** | 2.67* | 2.02* | 2.45* | 1.04 | 1.02 | 0.58 |
| ***Alp*** | 1.17 | 1.04 | 1.14 | -0.74 | -0.85 | -1.02 |
